# Omentum Free Flap Anastomosed to Arterial Bypass in Open Knee Dislocation: Case Report and Discussion

**Published:** 2015-06-11

**Authors:** Julien Pauchot, Emilie Ducroux, Grégoire Leclerc, Laurent Obert, Anne Pauline Sergent

**Affiliations:** ^a^Orthopedic, Traumatology, Plastic Reconstructive and Hand Surgery Unit; ^b^Vascular Surgery Unit, University Hospital of Besançon, University of Franche-Comte, Besançon, France

**Keywords:** knee dislocation, omentum, free flap, vascular bypass, microsurgery

## Abstract

**Objective:** To report on the original surgical management of a patient with severe trauma of both legs involving anastomosis of an omentum free flap with an emergency vascular bypass. **Methods:** After stabilization of the knee with an external fixator, a femoral-tibial bypass graft was performed to revascularize the leg with the contralateral great saphenous vein. Ten days later, an omentum free flap was used with an end-to-side arterial anastomosis between the right gastroepiploic artery and bypass graft to cover the loss of leg substance. **Discussion:** Anastomosis of a free flap with a single axis exposes the patient to risks of thrombosis and amputation. Lengthening of the arterial pedicle of the flap by venous graft or vascular loop might have allowed for avoidance of connection to the bypass. Nevertheless, the saphenous vein, generally used in these indications, was already harvested. The transitional anastomosis of the flap to the contralateral leg could not be considered because of the leg amputation. End-to-side anastomosis to the bypass presents many advantages: anastomosis with a healthy vessel without posttraumatic vascular disease, the superficial characteristics of the bypass, and lower incongruence of the thickness between the vessels compared with an anastomosis performed directly on the superficial femoral artery. **Conclusion:** A free flap anastomosed to an emergency arterial bypass is a rare situation, which is not without risk, but it is an option that is justified by its technical simplicity. However, it should only be considered in exceptional circumstances.

This study reports on the original surgical management of a patient with severe trauma of both legs involving anastomosis of an omentum free flap with an emergency vascular bypass.

## CASE REPORT

A 38-year-old man had both his lower limbs caught by an agitator-mixer, which required amputation of the lower third of the right leg. In addition, the left limb presented with an open dislocation of the left knee, ischemia of the leg, and a skin defect on the posterior aspect of the knee that was associated with third-degree burns, from caustic soda, covering 4% of the body surface.

The leg was immobilized with an external femorotibial external fixation, and the continuity of the posterior tibial and fibular nerves to the knee was checked intraoperatively. The greater saphenous vein was harvested from the contralateral lower limb, and a femoral-tibial bypass was performed to revascularize the leg. The patient underwent amputation of the middle third of the right leg. Prophylactic aponeurotomies were performed in the 3 compartments of the leg.

**Figure 1:**
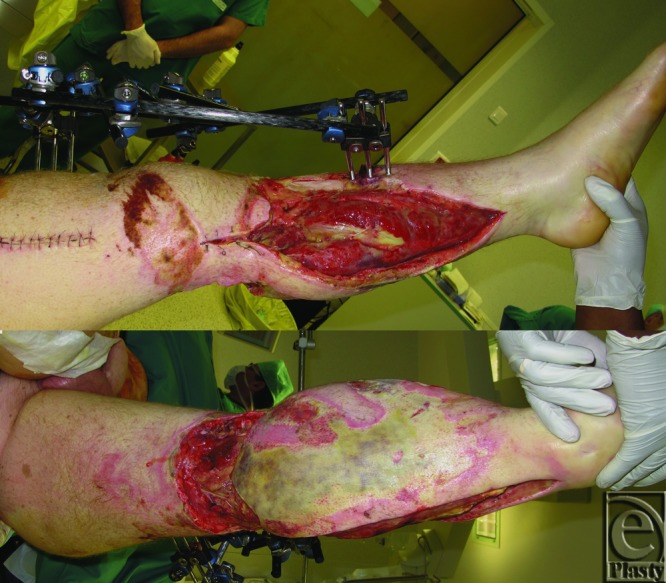
Cutaneous defect and aponeurotomies with bone, bypass, and knee joint exposition. Medial and posterior views.

On the 10th day, an omentum free flap was harvested with median laparotomy to treat the patient for exposure of the knee joint, upper part of the tibia, and vascular bypass at the surgical approach to the leg (Fig 1).

**Figure 2:**
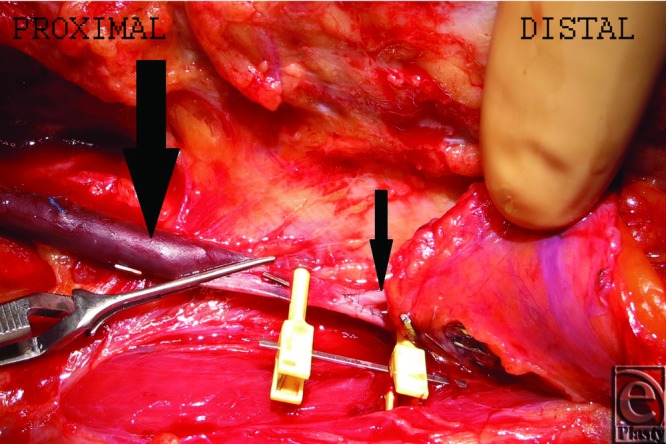
End-to-side arterial anastomosis between the right gastroepiploic artery (small arrow) and saphenous vein bypass graft (big arrow).

**Figure 3:**
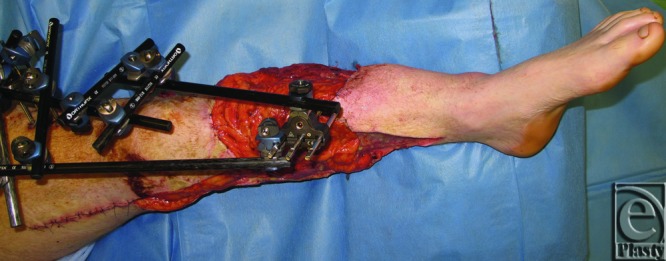
Immediate postoperative view.

**Figure 4:**
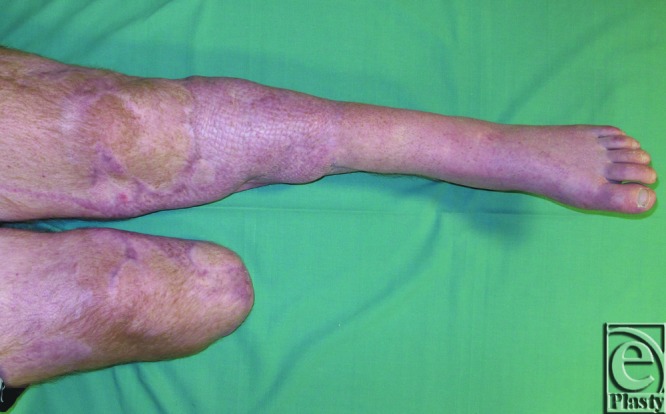
Clinical aspect after 1 year of follow-up.

The approach to the arterial bypass was revised in the thigh. Because of the ecchymotic aspect of the superficial femoral artery at a lower level of the bypass, an end-to-side arterial anastomosis was performed between the right gastroepiploic artery and the bypass saphenous vein (Figs 2 and 3) rather than with the superficial femoral artery. An end-to-end anastomosis between the right gastroepiploic vein and the internal saphenous vein of the left leg was then performed. An expanded split-thickness skin graft was performed 15 days later. The clinical features after 1 year of follow-up are shown in Figure 4.

## DISCUSSION

Knee dislocation results from high-energy trauma and exposes the patient to severe vascular and neural complications, but a large skin defect is unusual. The omentum free flap was selected because of its large surface of coverage, fineness such that it can match the type of substance lost, detersive properties, and granulating capacities. Kiricuta^1^ popularized the omentum free flap in the 1960s. Its average surface is 400 cm^2^, and it is vascularized by the right and left gastroepiploic arteries, which join along the greater curvature of the stomach. In its free flap form, it is usually harvested from the right gastroepiploic pedicle with vessels that have an average diameter of 2 mm. Its use in surgery to cover large losses of substance of the lower limbs has been reported in the literature in the treatment of chronic leg wounds^2^ or wounds with a traumatic origin.^3^ Interestingly, it is possible to split the free flap in 2 for each of the gastroepiploic arteries, covering bilateral losses of substance of the legs.^3^ The morbidity of the flap harvested by laparotomy is low, especially in the absence of a history of abdominal surgery, and it is reduced by laparoscopy.^4^

A latissimus dorsi free flap can be another option. However, loss of the most powerful adductor of the shoulder in a patient who would likely use crutches did not seem justified.

Among the other options for covering the loss of substance, negative pressure therapy was not selected because of the exposure and risk of compression of the bypass, risk of hemorrhage associated with anticoagulant treatment, and open dislocation of the knee.

Anastomosis of a free flap with a single axis is not specific to the lower limb, and it exposes the patient to risks of thrombosis. Lengthening of the arterial pedicle of the flap by venous graft or vascular loop^5^ might have allowed for avoidance of connection to the bypass at a distance from the lesions with a traumatic origin without increasing the risk of failure.^6^ Nevertheless, the saphenous vein, generally used in these indications, was already harvested, which would have justified the use of the cephalic or basilic veins.^7^ For the same reason, the transitional anastomosis of the flap to the contralateral leg could not be considered.^8^

Anastomosis of a free flap with an arterial bypass by vein has previously been reported by vascular surgeons, but its use has mainly been reported for chronic arterial ulcers of the lower limbs.^9^ The procedure is mainly used in desperate cases in which the risk of amputation is more strongly linked, such as flap graft failure, to the chronicity of the wounds rather than to the risk of bypass thrombosis.

However, although our decision was not without risk, the end-to-side anastomosis to the bypass seemed a more straightforward option for several reasons, including anastomosis with a healthy vessel without posttraumatic vascular disease, the superficial characteristics of the bypass, and lower incongruence of the thickness between the vessels compared with an anastomosis performed directly on the superficial femoral artery.

The onset of posttraumatic vascular disease occurs 1 week after the trauma and sometimes up to 12 cm above the lesion. As it is associated with perivascular fibrosis, spasms, and decreased thromboresistance, some authors do not recommend anastomosis of a free flap to a vessel after 1 week or at a distance of less than 10 cm from the traumatic lesion.^10^

Interestingly, the short healing time between bypass execution and exposure facilitated this approach. It is probable that a more delayed approach to the bypass would have been more difficult because of the healing process.

## CONCLUSION

A free flap anastomosed to an emergency arterial bypass is a rare situation, which is not without risk, but it is an option that is justified by its technical simplicity. However, it should only be considered in exceptional circumstances. The omentum free flap retains its indications and remains “the extreme flap in extreme situations.”

## References

[1] Kiricuta I (1963). [The use of the great omentum in the surgery of breast cancer]. Presse Med.

[2] Piano G, Massad MG, Amory DW Jr (1998). Omental transfer for salvage of the moribund lower extremity. Am Surg.

[3] Seitz IA, Siwinski P, Rioux-Forker D, Pavone L, Schechter LS (2014). Upper and lower limb salvage with omental free flaps: a long-term functional outcome analysis. Plast Reconstr Surg.

[4] Saltz R, Stowers R, Smith M, Gadacz TR (1993). Laparoscopically harvested omental free flap to cover a large soft tissue defect. Ann Surg.

[5] Silveira LF, Patricio JA (1993). Arteriovenous fistula with a saphenous long loop. Microsurgery.

[6] Bayramicli M, Tetik C, Sönmez A, Gürünlüoğlu R, Baltaci F (2002). Reliability of primary vein grafts in lower extremity free tissue transfers. Ann Plast Surg.

[7] Harris RW, Andros G, Dulawa LB, Oblath RW, Salles-Cunha SX, Apyan R (1984). Successful long-term limb salvage using cephalic vein bypass grafts. Ann Surg.

[8] Tropet Y, Brientini JM, Vichard P (1990). [Free-flap transfer of the omentum to the lower leg]. Ann Chir Plast Esthet.

[9] McCarthy WJ III, Matsumura JS, Fine NA, Dumanian GA, Pearce WH (1999). Combined arterial reconstruction and free tissue transfer for limb salvage. J Vasc Surg.

[10] Godina M (1986). Early microsurgical reconstruction of complex trauma of the extremities. Plast Reconstr Surg.

